# Capsule Endoscopy: A Cause of Late Small Bowel Obstruction and Perforation

**DOI:** 10.1155/2013/458108

**Published:** 2013-12-11

**Authors:** Anders Peter Skovsen, Jakob Burcharth, Stefan Kobbelgaard Burgdorf

**Affiliations:** Department of Surgery, Herlev Hospital, University of Copenhagen, Herlev Ringvej 75, 2730 Herlev, Denmark

## Abstract

*Case Report*. A 71-year-old man was admitted to the department of gastroenterology with diffuse abdominal pain. Through the previous 12 months, the patient had experienced episodes of vomiting and watery diarrhea of increasing intensity as well as weight loss. The patient was evaluated with ultrasound, MRI, and subsequently a capsule endoscopy. Six months later, the patient presented, and an abdominal CT-scan showed mechanical small bowel obstruction with suspicion of metallic foreign body and perforation. Laparotomy showed perforation, stenosis, and foreign body, approximately 5 cm from the ileocecal valve. A right hemicolectomy and distal ileectomy (60 cm) with an ileostomy were performed. On further inspection of resection, a capsule endoscope was found impacted in a stenosis. The ileostomy was later reversed without complications. *Conclusion*. It is important to be aware of the possibility of capsule retention, especially in patients with known or suspected Crohn's disease, due to the propensity of Crohn's disease to form stenosis of the bowel. In cases where a stenosis is suspected, it is warranted to perform a patency capsule swallow before subjecting the patient to a capsule endoscopy.

## 1. Introduction

Capsule endoscopy (CE) has been used since the year 2000, and over 600,000 CE have been performed until now [1, 2]. CE is useful in diagnosing small bowel conditions such as occult bleeding and possible inflammatory bowel disease (IBD) and is generally a secure and safe examination. However, complications can occur. Capsule retention is among the most serious complications and is defined as the capsule not having passed through the bowel within two weeks of ingestion [3]. Retention of the capsule is often seen by structural small bowel abnormalities such as ulcer, tumours, and narrow anastomoses [4]. So far no cases of capsule retention in normal small bowel have been reported. Only three case reports including a total of five patients have reported capsule retention leading to mechanical small bowel obstruction.

The aim of this case report is to present an atypical case of capsule retention leading to mechanical obstruction and small bowel perforation six months after ingestion of the capsule.

## 2. Case Report

A 71-year-old man was admitted to the department of gastroenterology with diffuse abdominal pain. Through the previous 12 months, the patient had experienced episodes of vomiting and watery diarrhea of increasing intensity as well as weight loss. Both abdominal ultrasound and MRI had shown signs of terminal ileitis. Despite also having undergone a full colonoscopy and gastroscopy without confirmation of inflammatory bowel disease, the patient was suspected of having Crohn's disease. Six months prior to admission, the patient had swallowed a capsule endoscope, and the image feed was deemed normal.

**Figure 1:**
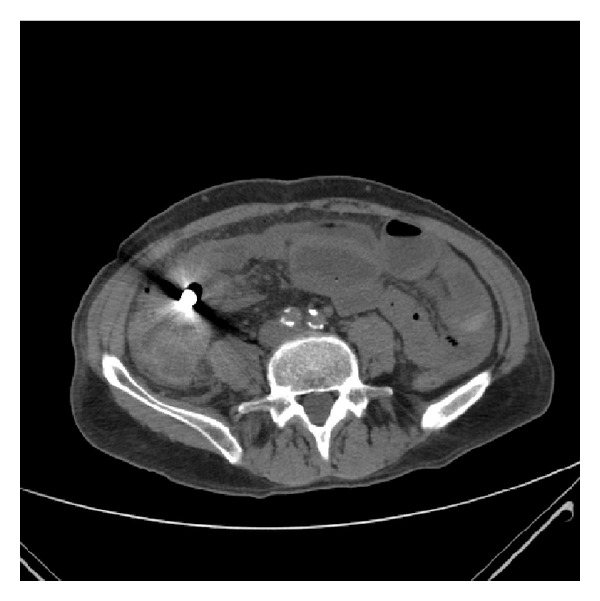
CT-abdomen showing small bowel obstruction, radiodense foreign body (capsule endoscope), and intraperitoneal air.

On presentation, an abdominal CT-scan was performed (Figure 1), showing mechanical small bowel obstruction with suspicion of metallic foreign body and perforation. Lab results showed C-reactive protein (CRP) of 204 mg/L.

**Figure 2:**
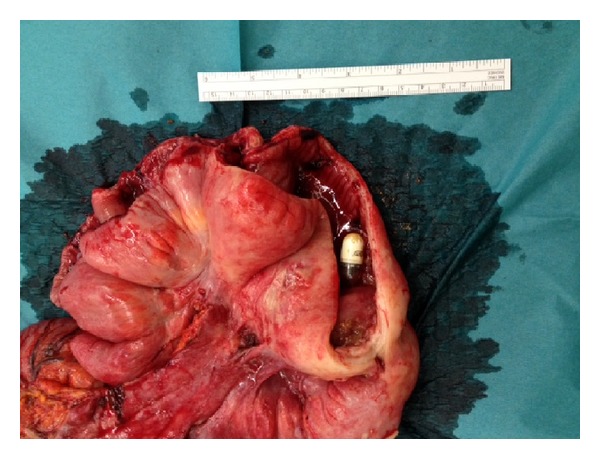
Photograph of bowel resection, cutup, showing capsule endoscope in situ.

The patient was taken to theater for a laparotomy. In the terminal ileum, approximately 5 cm from the ileocecal valve, a stenosis and a foreign body were found. Adhesions, chronic inflammation, and fibrinous cover characterized the area. A small perforation with localized fecal peritonitis was discovered near the ileocecal valve. A right hemicolectomy and distal ileectomy (60 cm) with an ileostomy were performed. On further inspection of resection, a capsule endoscope was found impacted in a stenosis (Figure 2). The stenosis was histologically verified as Crohn's disease. The ileostomy was later reversed without complications.

## 3. Discussion

Small bowel obstruction following retention of a capsule endoscope is very rare. We present a case where impaction of the retained capsule resulted in small bowel obstruction more than six months after ingestion.

Crohn's disease can affect the entire digestive tract. No gold standard exists in the diagnosis of the disease, and definitive diagnosis is often obtained by a combination of clinical, biochemical, radiological, endoscopic, and immunological findings. Capsule endoscopy is superior in assessing small bowel mucosal lesions compared to all other modalities, especially in the proximal and mid-small bowel [5]. Capsule retention is defined as having a capsule remaining in the digestive tract for a minimum of 2 weeks. Only surgical or endoscopic treatment has proven viable in removing a retained capsule. There are no data for successful medical treatment [6].

In a systematic review involving 22,840 procedures, 184 capsules were reported to be retained in 104 prospective studies and in 46 retrospective studies [3]. The pooled retention rate was 1.4%, which also has been reported by others [7]. Retention rates for confirmed Crohn's disease are reported as 5–13% [6].

It is important to be aware of the possibility of capsule retention, especially in patients with known or suspected Crohn's disease, due to the propensity of Crohn's disease to form stenosis of the bowel. In cases where a stenosis is suspected, it may be warranted to perform a patency capsule swallow before subjecting the patient to a capsule endoscopy.
